# Influence of Glenosphere and baseplate parameters on Glenoid bone strains in reverse shoulder Arthroplasty

**DOI:** 10.1186/s12891-019-2968-3

**Published:** 2019-12-05

**Authors:** Leo Pauzenberger, Cory Dwyer, Elifho Obopilwe, Michael D. Nowak, Mark Cote, Anthony A. Romeo, Augustus D. Mazzocca, Felix Dyrna

**Affiliations:** 1 Vienna Shoulder & Sports Clinic, Vienna, Austria; 2 Sports Surgery Clinic, Dublin, Ireland; 3 Department of Orthopaedic Surgery, University of Connecticut Health Center, Farmington, CT USA; 4 College of Engineering, Technology, and Architecture, University of Hartford, Hartford, West, CT USA; 5 Rothman Orthopaedic Institute, New York, NY USA; 6 Department of Orthopaedic Sports Medicine, Technical University Munich, Munich, Germany

**Keywords:** Reverse shoulder arthroplasty, Bone strains, Glenoid failure, Scapular notching, Glenosphere, Baseplate position, Biomechanics

## Abstract

**Background:**

Little is known about the strains at the glenoid near the bone-implant interface in reverse shoulder arthroplasty. The purpose of the current study was to evaluate the strains on the glenoid bone under a compressive load after implantation of three different sizes of metal-backed baseplates in either inferior or superior position in combination with three different sizes of glenospheres and three different glenosphere designs.

**Methods:**

Three sizes of baseplates (small, medium, large) were implanted in thirty-six paired human cadaveric scapulae either inferior, flush with the glenoid neck, or with a 5 mm superior offset. Glenospheres were available in three sizes (36 mm, 39 mm, 42 mm) and designs (standard, 4 mm lateralized, 2.5 mm inferiorized). Specimens were mounted in a servo-hydraulic testing apparatus at a 60° angle between the glenoid and actuator holding the humeral component. Four strain-gauge rosettes were placed around the glenoid rim to measure strains transferred to the scapular bone under a compressive load (750 N) relative to the various baseplate-glenosphere combinations. Following repeated compression, a load-to-failure test was performed.

**Results:**

Mean overall registered strains were 161με (range: − 1165 to 2347) at the inferior sensor, −2με (range: − 213 to 90) at the superior sensor, −95με (range: − 381 to 254) at the anterior sensor, and 13με (range: − 298 to 128) at the posterior sensor. Measured bone strains did not show any significant differences across tested baseplate and glenosphere design, size, or positioning combinations (*p > 0.05* for all sensors). Furthermore, linear regression analysis did not identify any of the evaluated parameters as an independent influential factor for strains (*p > 0.05* for all sensors). Mean load-at-failure was significantly higher in the group of inferior (3347.0 N ± 704.4 N) compared to superior (2763.8 N ± 927.8 N) positioned baseplates *(p = 0.046)*.

**Conclusion:**

Different baseplate positions, baseplate sizes, glenosphere sizes, and glenosphere design or various combinations of these parameters did not significantly influence the measured bone strains at the glenoid near the bone-implant interface in a contemporary reverse shoulder arthroplasty system.

**Level of evidence:**

Basic Science Study, Biomechanical Study.

## Background

Reverse shoulder arthroplasty has been proven to be an effective treatment for patients with cuff tear arthropathy or other degenerative diseases of the glenohumeral joint. Furthermore, reverse arthroplasty has been successfully used in cases of soft tissue or bone insufficiency as a revision or salvage option [1, 2]. However, glenoid failure remains a problem of reverse shoulder arthroplasty caused by mechanical and biological factors, most likely by a combination of anatomic and implant design aspects influencing mechanics, patient demands, available bone stock including its quality, initial fixation strength, and long-term stability ensured by secure osseous integration [3, 4].

It has previously been recognized that osseous integration of implants is promoted when loads are readily transferred to the cortical bone [5–8]. When these loads are transferred to the bone, it is strained and resists with a force called stress. These strains on the bone are commonly expressed as microstrains (με) [9]. While a certain amount of strain is important for native bone health and ingrowth of implants, non-physiological loads, either by exceeding the natural limits or deloading parts of the bone, can lead to acute fracture or implant loosening and failure over time. Consequently, these processes play an important role in implant design and longevity [9].

Especially throughout the history of reverse shoulder arthroplasty, many cycles of failures related to these concepts, subsequent expanding understanding, and design adaptions preceded its current triumph [3, 10]. To accompany an ever wider range of pathologies, soft tissue properties, and patient morphologies, various glenoid components and glenosphere designs have been introduced recently for reverse shoulder arthroplasty, but little is known about the strains developed around the glenoid components after implantation. Increased knowledge about these characteristics in contemporary arthroplasty systems could lead to future improvements of implants to increase the long-term survival rates and outcomes of reverse shoulder arthroplasty.

The purpose of the current study was to evaluate the strains on the glenoid bone under a compressive load after implantation of three different sizes of metal-backed baseplates in either inferior or superior position in combination with three different sizes of glenospheres and three different glenosphere designs. Our hypothesis was that glenoid bone strains do not differ between baseplate and glenosphere combinations.

## Methods

Thirty-six paired human cadaveric scapulae (age: 60.9 ± 7.5 years; bone mineral density: 0.476 ± 0.163 kg/m^3^; female, *n* = 19 - male, *n* = 17) were cleaned of all soft tissues, and allocated into three groups relative to their size. For this purpose, the scapulae were inspected by the senior surgeon (ADM) and allocated into the three groups according to the best fitting glenoid baseplate guide (small, *n* = 12; medium, *n* = 12; large, *n* = 12). Bone mineral density was measured (dual-energy x-ray absorptiometry) for all scapulae at a 1x1cm region (anterior to posterior) of the inferior third of the glenoid. There was no difference in bone mineral density between glenoid size (small, medium, large) or glenoid positioning groups (inferior vs. superior, during load-to-failure testing). All specimens were thawed overnight and experiments were performed at room temperature within the next 24 h. Specimens were obtained from Medcure Inc. (Portland, OR).

A current reverse arthroplasty system (Univers Reverse II, Arthrex, Naples, FL), used by the senior author in his clinical practice, was used for this study. The glenoid baseplate of this system features an anatomic baseplate shape, as opposed to a circular design, a central peg and screw (6.5 mm) combination, and two (inferior and superior) variable angle screws (4.5 mm). Baseplates of three sizes (small, medium, large; height: 32 mm–38 mm, width: 24.4.mm-30.4 mm, thickness: 4 mm) were implanted according to glenoid size in the three groups. In each group (*n* = 12), baseplates were either implanted in an inferior position (*n* = 6), flush with the inferior glenoid rim, or were placed in a superior position (*n* = 6), defined as 5 mm superior offset from the inferior glenoid rim. The offset was measured and marked using a digital caliper with 0.01 mm resolution (Mitutoyo, Japan).

All surgeries were performed by an experienced, fellowship trained shoulder surgeon (A.D.M.) to reduce performance bias. Similar to the actual in-vivo surgical technique, a guide wire was placed first to ensure correct positioning of the glenoid implants. The goal was to place the baseplate in 0 to 10° retroversion and neutral inclination according to the surgeon’s judgement and clinical practice. Following over-drilling the guide wire to fit the central peg (12.1 mm diameter), the glenoid surface was reamed down beyond the sclerotic zone, the baseplate was aligned with glenoid rotation, impacted, and fixed with a 6.5 mm diameter central screw (15 mm, 20 mm, 25 mm lengths). Two additional 4.5 mm locking screws (24 mm–48 mm length with 4 mm increments) were used at the superior and inferior position. The maximally possible screw lengths to avoid any bone perforation were chosen by measuring with a depth gauge. Specimens were trimmed and potted in custom boxes with the baseplate-surface parallel to the floor in all planes. Scapulae were then mounted in a servo-hydraulic mechanical testing apparatus (MTS, Eden Prairie, MN) to simulate a 60° glenohumeral angle between the glenoid and actuator holding the humeral components (neutral metal cup and 3 mm polyethylene liner) in a custom fixture, which were available in three different sizes (36 mm, 39 mm, 42 mm) corresponding to available glenospheres. The angle for testing was chosen according to previous studies, showing peak shear forces occurring at 60° of abduction [11, 12].

**Fig. 1:**
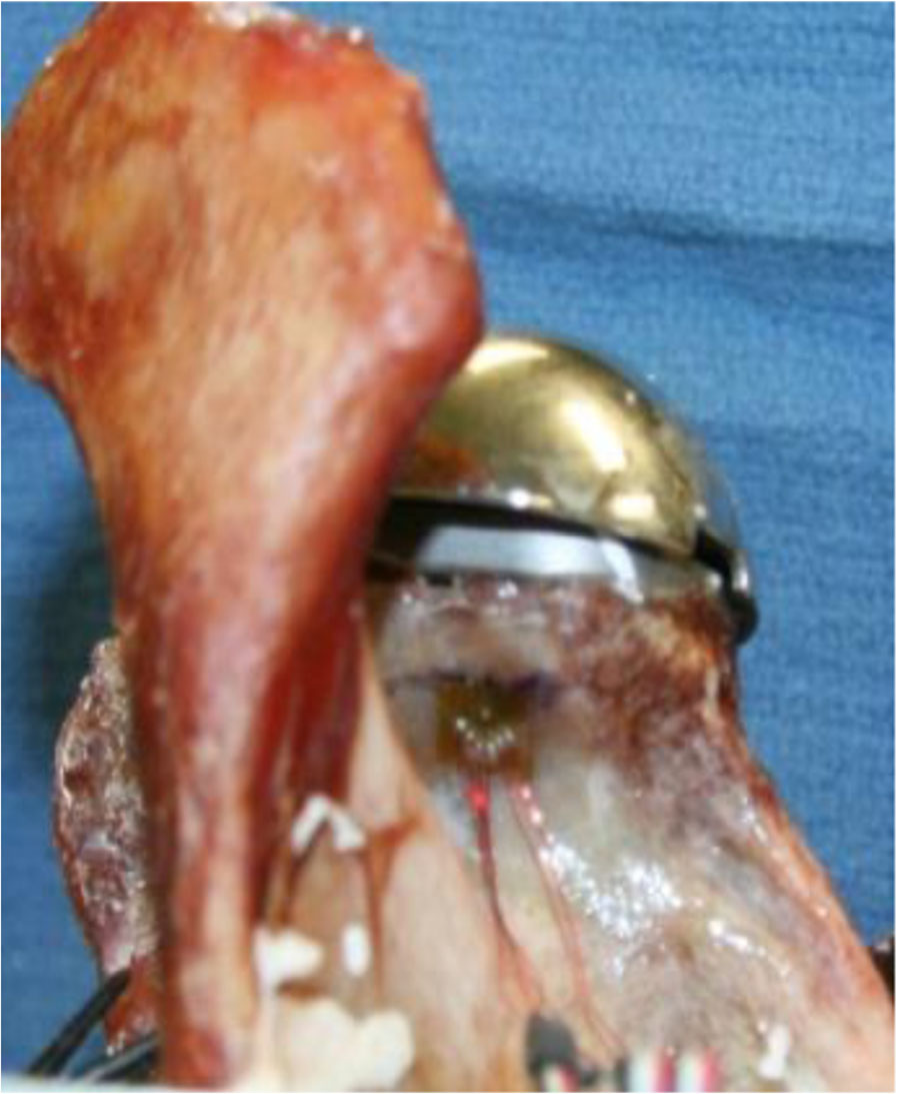
Superior strain gauge (Vishay, Raleigh, NC) at the 12 o’clock position, 5 mm medial to the reamed glenoid rim

Four stacked 0–45-90° rosette 350 Ohm strain gauges (Vishay, Raleigh, NC) were placed on the scapula 5 mm medial to the glenoid surface at the corresponding 12, 3, 6, and 9 o’clock positions. Strain gauges were fixed to the bone with a thin layer of cyanoacrylate adhesive (Loctite® 411, Henkel, Düsseldorf, Germany) and covered with tape for 24 h before testing. (Fig. 1) A 12-channel strain data recorder (Model 5100 Scanner) was used to log readings of all rosettes simultaneously at 20 Hz (StrainSmart 4.31 Software). Principal strain values were calculated for each rosette. The strain gauges were re-calibrated between each round of testing.

Three pilot studies were performed to verify the accuracy of the strain gauges and get an understanding of the interaction between strain and load values. First, the accuracy and reproducibility of the strain gauges on a homogeneous surface were tested. Two strain gauges were placed on a 1″ polyvinylchloride pipe and a compressive load of 750 N was applied repeatedly (five times) in axial direction on a mechanical testing system (MTS, Eden Prairie, MN), where the strain gauges provided a consistent strain output (sensor 1: 219 ± 4.4, sensor 2: 220 ± 2.2). Average error between the sensors was 0.99 (0.45%) in reading strain under the repeatedly applied 750 N load. Second, to ensure that sequential measurements with different glenospheres were not influenced by the manual exchange of components, a pilot series of repeated strain measurements was performed. Between each trial, the glenosphere was removed and repositioned on the baseplate. The strain gauges were re-calibrated between each repeated measurement and strain values were compared between trials. This process revealed consistent strain readings with an average error of 0.50 (1.16%) across repeated trials and all strain gauge positions.

Third, in an effort to make the concept of strain easier to understand and put into context, strains during load-to-failure were evaluated in order to compare strains encountered during activities of daily living with what failure strain is. A load-to-failure test (*n* = 12) was conducted by applying a constant compressive force at a rate of 0.2 mm/sec and logging the peak load. Peak loads at failure ranged from 1537.65 N to 4150.30 N, while principal strains at failure were 6087.25 ± 2189.19 in this small pilot cohort. This last pilot study revealed that increasing strain values were consistent with increasing macroscopically visible osseous deformity and location of failure. In combination, these results suggested that the strain gauges were able to adequately register supraphysiological bone strains indicative of component or bone failure.

### Testing sequence

**Fig. 2:**
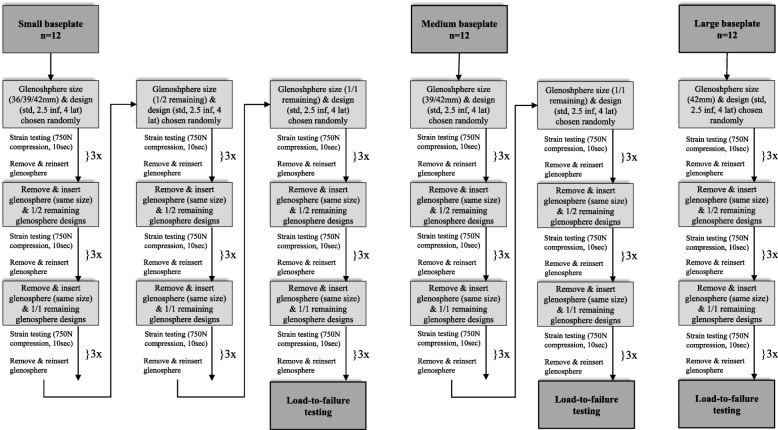
Flowchart illustrating the testing sequence. *Std* Standard glenosphere, *2.5 Inf* 2.5 mm inferiorized glenosphere, *4 Lat* 4 mm lateralized glenosphere

**Fig. 3:**
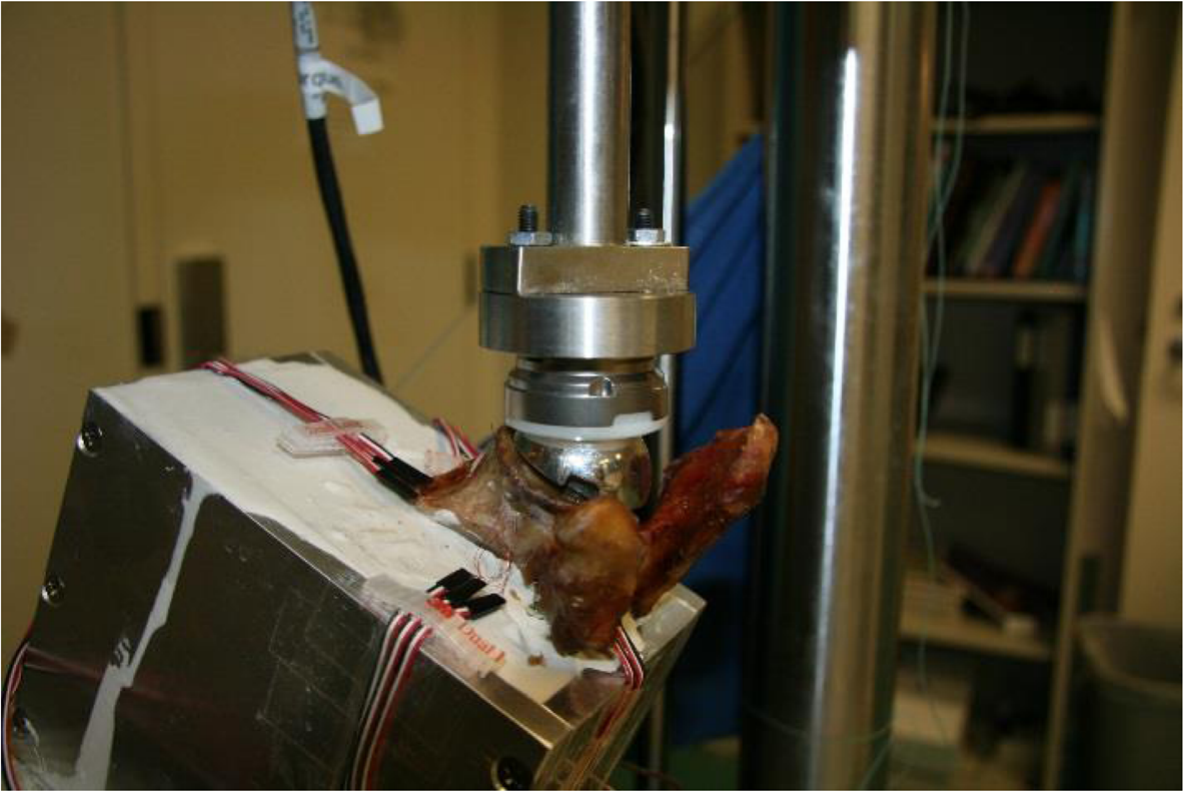
Biomechanical testing setup showing the mounted scapula with implanted glenoidal components, strain gauges around the glenoid rim, and the humeral component including a polyethylene cup attached via a custom fixation to the actuator

Three different glenosphere sizes (36 mm, 39 mm, 42 mm diameter) were available with three different designs each (standard, 2.4 mm inferior offset, 4 mm lateralized). The sequence for testing was chosen at random for different glenosphere sizes as well as designs to avoid any possible bias from micromotions caused by specific baseplate-glenosphere combinations. The glenosphere was placed on the baseplate and secured with a single hammer tap using a dedicated impactor. A compressive load of 100 N was applied for 60 s to center the glenosphere under the humeral component. Then a load of 750 N, which has been used in various prior studies [2, 11–15], was applied for 10 s to register bone strains. The glenospheres were then removed, re-inserted, and the testing sequence was repeated for a total of three times. Mean values were calculated for further statistical analysis. After completion of three test runs, the next glenosphere design was chosen randomly and the process was repeated until all glenosphere types were tested. Then the humeral components were removed and the next size of glenosphere-humeral cup combination was mounted for further testing. According to the possible glenospheres that could be used with the various baseplate sizes, this resulted in a total of 27 test runs for small, 18 for medium, and 9 for large baseplates, respectively (Fig. 2.). The incidence of abutment of the humeral component at the scapular neck was documented for all baseplate-glenosphere-positioning combinations. In case of abutment, the subsequently obtained strain values were excluded from statistical analysis under the assumption that the inferior strain gauge becomes damaged by the engaging humeral component. Following strain testing, a lateralized glenosphere (to avoid abutment as mode of failure) was mounted in the size of the baseplate and a load-to-failure test was performed by applying a constant compressive force at a rate of 0.2 mm/sec while documenting peak load at bone or component failure as well as the mode of failure. (Testworks 4, Eden Prairie, MN). The biomechanical testing setup is depicted in Fig. 3.

### Statistical analysis

Descriptive statistics were used to present demographical data of specimens. One-way analysis of variance (ANOVA) with Tukey post-hoc test was used to evaluate differences between three or more independent groups. Linear mixed effects regression was used to generate mean values and 95% confidence intervals for principal strain across conditions (baseplate size, glenosphere size, glenosphere design, and baseplate position), with a random effect at the specimen level. This approach accounted for the correlation between shoulders as specimens were used in multiple conditions. A custom matrix was created to illustrate the incidence of humeral component abutment based on the various baseplate-glenosphere combinations with different baseplate positions. The Chi^2^-test was used to compare the incidence of abutment between baseplate positioning and baseplate-glenosphere combinations. Confidence intervals of proportions or counts were calculated according to the modified Wald method [16]. The alpha level for all statistics was set at 0.05. All statistical analysis was performed using Stata 14 (StataCorp. 2015. Stata Statistical Software: Release 14. College Station, TX: StataCorp LP).

## Results

### Strains

**Fig. 4:**
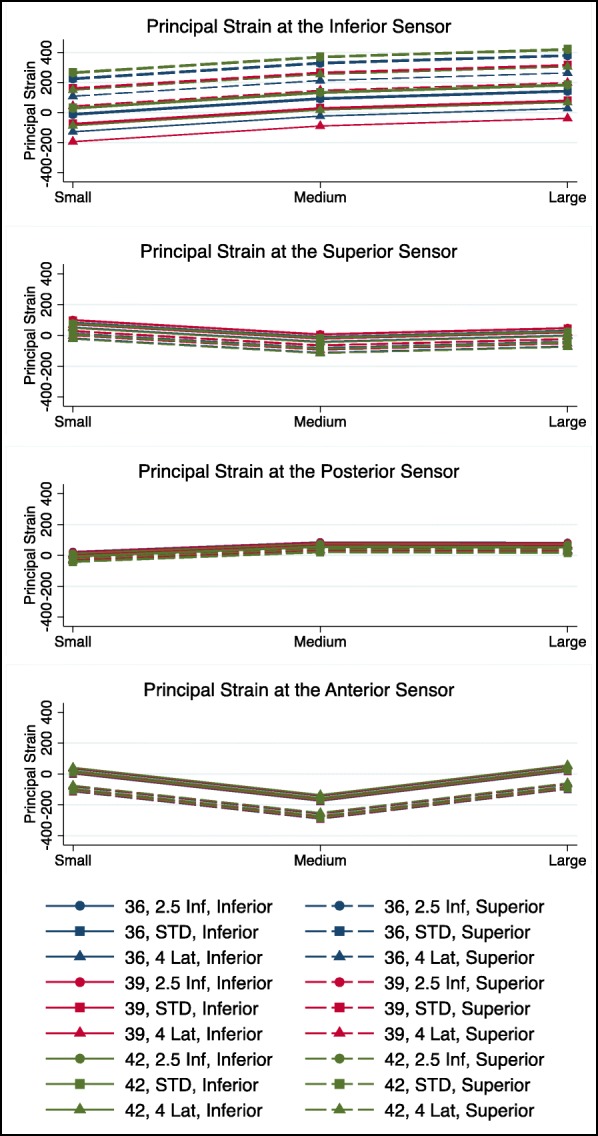
Line diagram illustrating principal glenoidal bone strains registered at the inferior, superior, posterior and anterior strain gauges during compressive loading relative to the various baseplate sizes, glenosphere sizes, and glenosphere designs. *Std* Standard glenosphere, *2.5 Inf* 2.5 mm inferiorized glenosphere, *4 Lat* 4 mm lateralized glenosphere

Discounting specimens discarded due to abutment of the humeral component with the scapular neck or the inferior strain gauge, respectively, 83.8% of measurements could be included in the strain analyses. Mean overall registered strains were 161με (range: − 1165 to 2347) at the inferior sensor, −2με (range: − 213 to 90) at the superior sensor, −95με (range: − 381 to 254) at the anterior sensor, and 13με (range: − 298 to 128) at the posterior sensor. When the baseplate was positioned inferior and flush with the glenoid, mean strains were 26με (range: − 405 to 244) at the inferior, 26με (range: − 1 to 66) at the superior, −138με (range: − 302 to 81) at the anterior, and 39με (range: − 46 to 128) at the posterior sensor, respectively. In superior placed baseplates, mean strains were 298με (range: − 1165 to 2347), −31με (range: − 213 to 90) superior, −51με (range: − 381 to 254) anterior, and -13με (range: − 298 to 71) posterior (Fig. 4.). However, the measured bone strains at the glenoid near the bone-implant interface, did not show any significant differences across tested baseplate and glenosphere design, size, or positioning combinations (*p > 0.05* for all sensors). Furthermore, linear regression analysis did not identify any of the evaluated parameters as an independent influential factor for strains (*p > 0.05* for all sensors, Table 1.).

**Table 1:** Glenoid bone strains during compressive loading

	Inferior sensor	Superior sensor	Anterior sensor	Posterior sensor
Baseplate size				
*Small*	43.3με (− 247.6 to 334.1)	42.8με (−7.4 to 93.1)	−38.5με (− 184.2 to 128.2)	− 7.4με (− 87.3 to 67.0)
*Medium*	147.6με (− 276.6 to 571.8)	−50.4με (− 120.5 to 19.6)	−153.8με (− 370.8 to − 40.0)	113.7με (− 78.9 to 183.7)
*Large*	198.4με (− 331.1 to 727.8)	−9.4με − 69.6 to 50.9	−7.5με (− 179.2 to 153.9)	51.5με (−25.6 to 123.2)
Glenosphere size				
*36*	101.5με (− 399.7 to 602.7)	2.0με (− 40.2 to 44.2)	−51.4με (− 188.3 to 19.7)	6.7με (−41.3 to 93.4)
*39*	35.8με (− 310.4 to 382.1)	19.1με (−18.3 to 56.6)	−93.4με (− 181.9 to 20.5)	19.4με (−37.7 to 84.0)
*42*	142.1με (− 147.5 to 431.8)	−3.2με (− 39.9 to 33.6)	−70.9με − 177.4 to 24.1	26.3με (−45.6 to 74.4)
Glenosphere design				
*Std*	149.0με (− 217.8 to 515.9)	14.1με (− 24.5 to 52.8)	− 158.7με (− 197.6 to 6.0)	40.4με (− 53.7 to 71.4)
*2.5 Inf*	140.3με (− 181.7 to 462.3)	18.3με (− 18.9 to 55.6)	−75.6με (− 184.4 to 17.6)	60.7με (− 29.3 to 92.0)
*4 Lat*	29.0με (− 281.2 to 339.2)	−13.1με (− 49.9 to 23.7)	−80.4με (− 164.4 to 37.3)	55.0με (−44.7 to 75.4)
Baseplate position				
*Inferior*	−0.4με (− 275.2 to 274.4)	37.0με (− 6.6 to 80.5)	−147.3με (− 137.3 to 82.5)	50.7με (−33.7 to 102.8)
*Superior*	236.5με (− 81.9 to 554.8)	−34.0με (− 79.0 to 10.9)	−36.5με (− 255.0 to − 32.2)	6.0με (− 71.7 to 72.1)

Presented values (microstrains; με) for each variable (e.g. baseplate size: small, etc.) at each sensor represent the mean strain value measured across all possible testing combinations of baseplate size, glenosphere size, glenosphere design, and baseplate position. Data is presented as mean principal strains and 95% confidence intervals. *Std* Standard glenosphere, *2.5 Inf* 2.5 mm inferiorized glenosphere, *4 Lat* 4 mm lateralized glenosphere. Measured strains did not show any significant differences across tested baseplate and glenosphere design, size, or positioning combinations (*p* > 0.05 for all sensors). Furthermore, linear regression analysis did not identify any of the evaluated parameters as an independent influential factor for strains (*p* > 0.05 for all sensors)

**Fig. 5:**
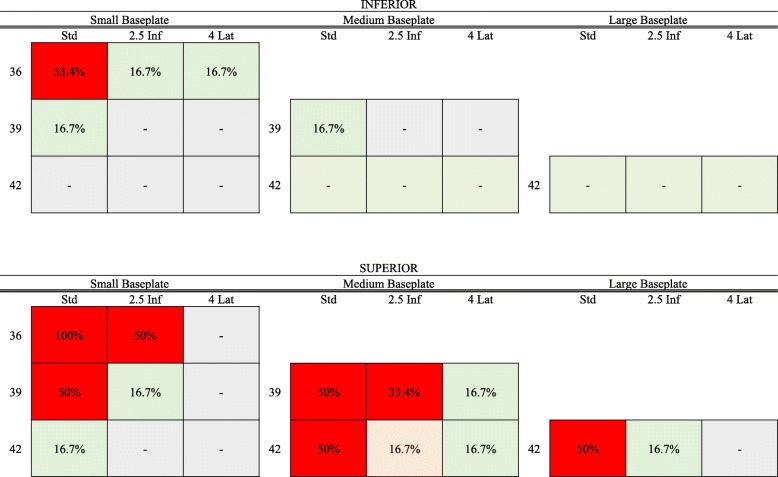
Custom matrix illustrating the incidence of abutment between humeral component and scapular relative to all available baseplate-glenosphere combinations, as well as inferior and superior baseplate position. *Std* Standard glenosphere, *2.5 Inf* 2.5 mm inferiorized glenosphere, *4 Lat* 4 mm lateralized glenosphere

Abutment of the humeral component at the scapular neck occurred in 16.2% of all cases. Significantly more often found in the superior positioning compared to inferior positioned (5.6% vs. 26.9%; *p < 0.001)* baseplates, the highest incidence was seen in the combination of a small, superior positioned baseplate and a 36 mm standard glenosphere (100%; *p < 0.001*). The incidence of this abutment and subsequent exclusion from quantitative strain analyses for all tested implant combinations is illustrated in Fig. 5.

**Fig. 6:**
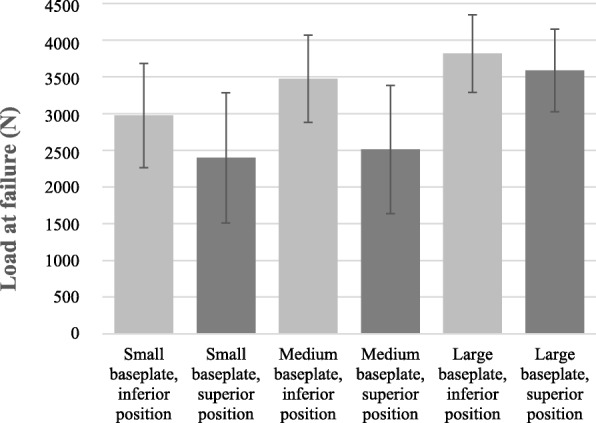
Peak load at failure under a compressive load according to baseplate size and position. All load-to-failure tests were performed with a glenosphere corresponding to the size of the baseplate (e.g. small glenosphere with small baseplate, etc.). Value are presented as means ± SDs

Mean load-to-failure across all baseplate and glenosphere combinations was 3061.9 N ± 863.6 N overall (Fig. 6.). The mean peak load at failure of 3347.0 N ± 704.4 N was significantly higher in the group of inferior positioned baseplates compared to 2763.8 N ± 927.8 N in the group of superior positioned components (*p* = 0.046). The main modes of failure were inferior glenoid impression fracture in the inferior positioning group and superior blow-out scapular fracture in the superior placement group.

## Discussion

The most important findings of the current study were that different baseplate positions, baseplate sizes, glenosphere sizes, and glenosphere design or various combinations of these parameters did not significantly influence the measured bone strains at the glenoid near the bone-implant interface.

It is well documented that strains exerted on the bone have pivotal effects on the bone remodeling process. It was also shown that mechanical strains promote bone ingrowth and, thus, enhance fixation of cementless implants [6, 7, 9, 17–19]. However, excessive as well as insufficiently high strains affecting the bone around implants could potentially lead to disruption of the physiological bone remodeling process, failure of osseo-integration, and ultimately result in component loosening. The highest strains are usually concentrated around implant and cortical bone, whereas the strains on the cortical bone increase with decreasing quality of surrounding cancellous bone [9, 20–22].

Especially in older adults, who are the recipients of most implants, the bone is unable to react adequately to excessive strains causing local microdamage, which can result in increased bone remodeling around an implant and ultimately loosening [9]. It has been established that such excessive microdamage of the bone occurs when strains exceed 4000με under compressive forces and 2500με under tensile forces [9, 18, 19, 23]. The results of the present study showed that the registered bone strains around the bone-implant interface at the glenoid neck stayed well within this physiological zone, independent of baseplate size or position, glenosphere size, and glenosphere design. This suggests that all baseplate-glenosphere combinations may provide an adequate biomechanical environment for secure fixation and osseous integration [9, 18, 19, 23].

Scapular notching, which has been implicated as a cause for radiolucent lines, decreased clinical outcome, and reverse arthroplasty failure, has been reported to occur as frequently as 70% [24]. Our current study showed that certain baseplate-glenosphere combinations, especially those including a small baseplate, exhibited a high risk of abutment between the humeral component and the scapular neck, which was further increased by superior positioning of the baseplate. Although this is generally in accordance with both biomechanical [25] and clinical results [24] of previous studies, the testing setup was not optimized for evaluation of scapular notching and various determining factors (e.g. scapular neck anatomy) were beyond the scope of the current study.

The incidence of aseptic glenoid loosening of contemporary reverse arthroplasty systems has been reported between 0 and 12%, with an average of 5%, in the short- and mid-term, while recent study projected a ten-year glenoid loosening rate of 16% [12, 26]. Even under a load of 750 N in 60° of glenohumeral abduction, which represents a worst-case scenario for activities of daily living, bone strains did not register outside of the proposed safe zone of 2500με under tensile and 4000με under compressive forces, respectively [9, 18, 19, 23]. These results indicate that independent of the used components and baseplate position on the glenoid, the used reverse arthroplasty system seems to provide secure fixation supporting osseous integration and, thus, in theory longterm survival with low risk of glenoid failure.

The current study has certain limitations that have to be considered when interpreting its results. There were no significant differences in bone strains found among the different component and positioning combinations. None of the evaluated variables could be retained in the regression model as independent factor for influencing bone strains. The irregular geometrical shape of the glenoid in conjunction with natural variations in component positioning and anatomy might have prevented the identification of distinct bone strain patterns. Despite including 36 cadaveric specimens, the study potentially could have been underpowered to identify small effect sizes. Nonetheless, the strains registered in the current study stayed well within the physiological range independent of baseplate-glenosphere combinations. The current investigation was performed in a static setting, without simulating movement through the range of motion of the shoulder joint. However, the chosen angle of abduction for testing has been previously identified as the position in which the highest shear forces are occurring [11, 12], thus, representing a worst-case scenario. Finally, the results from this study are specific to the used reverse shoulder arthroplasty implant system and cannot be unconditionally transferred to other reverse shoulder arthroplasty implants.

## Conclusion

Different baseplate positions, baseplate sizes, glenosphere sizes, and glenosphere design or various combinations of these parameters did not significantly influence the measured bone strains at the glenoid near the bone-implant interface in a contemporary reverse shoulder arthroplasty system.

## Data Availability

The datasets generated and analyzed during the current study are not publicly available, but are available as de-identified data sheet from the corresponding author on reasonable request.
